# N-benzyl-N-methyldecan-1-amine and its derivative mitigate 2,4- dinitrobenzenesulfonic acid-induced colitis and collagen-induced rheumatoid arthritis

**DOI:** 10.3389/fphar.2023.1095955

**Published:** 2023-04-20

**Authors:** Ji Eun Kim, Changyu Kang, Phatcharaporn Budluang, Natpaphan Yawut, Il-Rae Cho, Yun Ju Choi, Jaejeong Kim, Sanghyun Ju, Beomgu Lee, Dong Hyun Sohn, Hyung-Soon Yim, Kyeong Won Lee, Jinsol Han, Youngmi Jung, Ho Young Kang, Jin Kyoon Park, Yunjin Jung, Dae Youn Hwang, Young-Hwa Chung

**Affiliations:** ^1^ Department of Biomaterials Science (BK21 FOUR Program), College of Natural Resources and Life Science, Pusan National University, Miryang, Republic of Korea; ^2^ College of Pharmacy, Pusan National University, Busan, Republic of Korea; ^3^ Department of Cogno-Mechatronics Engineering, Optomechatronics Research Institute, Pusan National University, Busan, Republic of Korea; ^4^ Hayoung Meditech Inc., Busan, Republic of Korea; ^5^ Department of Microbiology and Immunology, College of Medicine, Pusan National University, Yangsan, Republic of Korea; ^6^ Korea Institute of Ocean Science and Technology, Marine Biotechnology Research Center, Busan, Republic of Korea; ^7^ Department of Biological Science, Department of Integrated Biological Science, Pusan National University, Busan, Republic of Korea; ^8^ Department of Microbiology, Pusan National University, Busan, Republic of Korea; ^9^ Department of Chemistry and Chemistry Institute of Functional Materials, Pusan National University, Busan, Republic of Korea

**Keywords:** RA: rheumatoid arthritis, N-benzyl-N-methyldecane-1-amine, rat colitis, anti-inflammatory activity, anti-oxidative activity

## Abstract

As our previous study revealed that *N*-benzyl-*N*-methyldecan-1-amine (BMDA), a new molecule originated from *Allium sativum*, exhibits anti-neoplastic activities, we herein explored other functions of the compound and its derivative [decyl-(4-methoxy-benzyl)-methyl-amine; DMMA] including anti-inflammatory and anti-oxidative activities. Pretreatment of THP-1 cells with BMDA or DMMA inhibited tumor necrosis factor (TNF)-α and interleukin (IL)-1β production, and blocked c-jun terminal kinase (JNK), p38 mitogen-activated protein kinase (MAPK), MAPKAP kinase (MK)2 and NF-κΒ inflammatory signaling during LPS stimulation. Rectal treatment with BMDA or DMMA reduced the severity of colitis in 2,4-dinitrobenzenesulfonic acid (DNBS)-treated rat. Consistently, administration of the compounds decreased myeloperoxidase **(**MPO) activity (representing neutrophil infiltration in colonic mucosa), production of inflammatory mediators such as cytokine-induced neutrophil chemoattractant (CINC)-3 and TNF-α, and activation of JNK and p38 MAPK in the colon tissues. In addition, oral administration of these compounds ameliorated collagen-induced rheumatoid arthritis (RA) in mice. The treatment diminished the levels of inflammatory cytokine transcripts, and protected connective tissues through the expression of anti-oxidation proteins such as nuclear factor erythroid-related factor (Nrf)2 and heme oxygenase (HO)1. Additionally, aspartate aminotransferase (AST) and alanine aminotransferase (ALT) levels did not differ between the BMDA- or DMMA-treated and control animals, indicating that the compounds do not possess liver toxicity. Taken together, these findings propose that BMDA and DMMA could be used as new drugs for curing inflammatory bowel disease (IBD) and RA.

## Introduction

Garlic (*Allium sativum*) possesses not only anticancer activity but also anti-inflammatory properties. The bioactive components of garlic have been largely classified in two groups: water-soluble and oil-soluble organosulfur composites. The water-soluble organosulfur compounds include *S*-allylmercaptocysteine, *S*-allyl cysteine, and allicin while lipid-soluble organosulfur compounds comprise ajoene, diallyl trisulfide diallyl disulfide, and diallyl sulfide (Kim et al., 2016; Zhang et al., 2020). For example, allicin, a mjor ingredient of garlic, downregulates HIF-1α leading to the enhanced sensitization to cisplatin in non-small cell lung cancer (Pandey et al., 2020) and activates Nrf2 signaling, resulting in decrease of LPS-mediated inflammation in human umbilical vein endothelial cells (Zhang et al., 2017). As a lipid-soluble organosulfur compound, diallyl trisulfide inhibits cancer stem cell phenotypes by hindering the action of Forkhead box Q1 and also suppresses LPS-induced inflammation through the NF-κB pathway (Lee et al., 2018).

*N*-Benzyl-*N*-methyldecan-1-amine (BMDA), recently separated from garlic extracts, arrests cells at G2/M phase in U937 human leukemia cells (Jeong et al., 2014). The result is attributed to the reduced levels of cyclin-dependent kinase(Cdk) 1 and 2, and conversely the enhanced expression of p21 Cdk inhibitor in the absence of p53 (Jeong et al., 2014). In addition, our previous study showed that BMDA, which is synthesized by an organic chemical method rather than extraction from raw garlic, inhibits a novel oncogene-induced TGF-β signaling through Akt-ERK1/2 and β-catenin pathways, leading to the prevention of cancer stem cell-like phenotypes (Kaowinn et al., 2018).

Inflammatory bowel disease (IBD) including Crohn’s disease and ulcerative colitis displays severe inflammatory lesions in the gastrointestinal tract (Abraham and Cho, 2009). It has been reported that the mucosal inflammation is caused by the dysregulated immune response to gut microflora besides environmental and genetic factors, although the etiology of IBD remains undetermined (Abraham and Cho, 2009; Uhlig and Powrie, 2018). Pharmacotherapies using chemical drugs, such as aminosalicylates, glucocorticoids, the peroxisome proliferator-activated receptor ligands, and biological drugs including anti-integrin and anti-tumor necrosis factor antibodies, have been applied for the treatment of IBD (Triantafillidis et al., 2011; Rath et al., 2018). However, we face great challenges to cure IBD due to low efficacy, resistance to the chemical drugs, and adverse side effects (Pithadia and Jain, 2011).

Rheumatoid arthritis (RA), a systematic chronic inflammatory disease, exhibits destruction of cartilage and bone in the hand and foot in which infiltration of inflammatory immune cells, synovial hyperplasia, and pannus formation were manifested although the etiology and pathogenesis remains to be thoroughly investigated (Noss and Brenner, 2008; Huang et al., 2021). Treatment paradigms have shifted from non-steroid anti-inflammatory drugs for an initial stage of RA to the administration of anti-TNF-α antibodies or JNK inhibitors for a late stage of RA (Demoruelle and Deane, 2012; Smolen et al., 2017). However, these treatments for the progressed RA also exhibit adverse side effects, including vulnerable infection of *Mycobacterium tuberculosis*, cytomegalovirus, heart failure, and malignancy (Hyrich et al., 2004).

In this study, we explore whether BMDA, a novel small compound derived from garlic, and its derivative DMMA, possess anti-inflammatory and anti-oxidative effects that can alleviate the pathological progression of colitis and RA. We first document that rectal treatment of colitis with BMDA or DMMA in DNBS-treated rat, an acute inflammatory disease model, and oral treatment with these small molecules in collagen-induced RA, a chronic inflammatory disease model, relieve the inflammatory process of the diseases.

## Materials and methods

### Antibodies and reagents

Antibodies against JNK (Cat. No; 9252s), p38 MAPK (Cat. No; 8690s), MK2 (Cat. No; 3042s), NF-κB (Cat. No; 4764) and their phospho-specific antibodies (Cat. No; 9255s, 9216s, 3316s, and 3033s, respectively) were acquired from Cell Signaling Technology (Danvers, MA, United States). Anti-HO1(Cat. No; sc-390991), - Nrf2 (Cat. No; sc-365949), -TNF-α (Cat. No; sc-52746), and -β-actin (Cat. No; sc-8432) antibodies purchased from Santa Cruz Biotechnology (Santa Cruz, CA, United States) were used to detect expression of the corresponding proteins. Lipopolysaccharide (LPS) and 2,4-dinitrobenzenesulfonic acid were acquired from Merck (St. Louis, MO, United States). Complete Freund’s adjuvant (CFA), incomplete Freund’s adjuvant (IFA), and bovine collagen type 2 obtained from Chondrex (Redmond, WA, United States) were utilized for induction of RA.

### Cell culture

Human monocytic THP-1 cells obtained from Korean Cell Bank (Seoul, Korea) were maintained in RPMI-1640 including 10% fetal bovine serum, 100 units/mL penicillin, and 100 μg/mL streptomycin at 37°C in a 5% CO_2_ atmosphere.

### Animal

For the care and use laboratory animals, animal experiments were carried out in consonance with the Laboratory Animal Resources Guide. The Pusan National University Animal Care and Use Committee approved protocols of the animal study related to rat colitis and RA (PNU-2021-3068 for rat colitis and PNU-2021-3014 for RA). Sprague Dawley (SD) rats (250–260 g, 7 weeks old, 20 male) and DBA/1 mice (22–25 g, 6 weeks old, 24 male) acquired from Samtako Biokorea (Ohsan, Korea) and Orient Bio Inc. (Seongnam, Korea) were used, respectively. They were kept under specific pathogen-free conditions (a 12-h interval cycle for lighting at 23 ± 2°C temperature and 50%–55% humidity) and free accessed to tap water and food.

### Synthesis of BMDA and DMMA

As previously described (Kaowinn et al., 2018), BMDA was synthesized with *N*-benzylmethylamine and decanal as starting materials using the reductive amination method (Molecules and Materials, Daejeon, Korea). DMMA was also synthesized with 1-(4-methoxyphenyl)-*N*-methylmethanamine and decanal using the same method (Molecules and Materials). The structures and molecular weights of the synthesized molecules were confirmed using H^1^-NMR and liquid chromatography-mass spectrometry (LCMS). ^1^H NMR (500 MHz, CDCl_3_) of BMDA was as follows: δ_H_ 7.41–7.19 (m, 5H), 3.50 (s, 2H), 2.37 (dd, *J* = 18.0, 10.4 Hz, 2H), 2.21 (s, 3H), 1.53 (dd, *J* = 14.3, 7.1 Hz, 2H), 1.39–1.20 (m, 14H), 0.91 (t, *J* = 6.9 Hz, 3H) ppm, and its molecular weight was also confirmed by LCMS (ESI+): *m/z* = 262.4 [M + H]^+^. ^1^H NMR (500 MHz, CDCl_3_) of DMMA was as follows: δ_H_ 7.24 (d, *J* = 8.6 Hz, 2H), 6.88 (d, *J* = 8.7 Hz, 2H), 3.83 (s, 3H), 3.45 (s, 2H), 2.40–2.33 (m, 2H), 2.19 (s, 3H), 1.52 (d, *J* = 7.2 Hz, 2H), 1.28 (s, 14H), 0.91 (t, *J* = 6.9 Hz, 3H) ppm; LCMS (ESI+): *m/z* = 292.5 [M + H]^+^.

### Induction of colitis in rats

As previously described (Kim et al., 2019), experimental colitis was generated in rats. Shortly, SD rats (male, 250–260 g) were starved for 24 h, except for tap water before the induction of colitis. Isoflurane was used for a light anesthesia of the rats. The rats were rectally inserted into the colon with a rubber cannula (2 mm OD). DNBS dissolved in 50% aqueous ethanol (48 mg, 0.4 mL^-1^) was interfused into the colon with the rubber cannula for 3 consecutive days. According to previously reported criteria, colonic damage score (CDS) was analyzed (Hong et al., 2012). The CDS assessment was performed by four independent observers blinded to the treatment conditions.

### Type II bovine collagen-induced rheumatoid arthritis (RA) in mice

Experimental RA was employed in mice as previously described (Kang et al., 2018). Bovine type II collagen (100 μg) mingled with an equal volume of CFA was introduced to DBA/1 mice (male, 7-8 weeks old) at the tail base of the animal. Day 0 indicates the day of the first immunization. On day 18 after the first immunization, the mice were augmented for immunity with an equal amount of the same collagen emulsified with 1:1 (v.v^−1^) IFA at the subcutaneous tail base and maintained for additional 2 weeks. Thickness of the paw at the widest point of the ankle joint was assessed using an electric caliper. All mice of subset groups were sacrificed in a CO_2_ gas chamber at 45 days after the first immunization.

### Hematoxylin and eosin staining analysis

The rear paws of DBA/1 mice were decalcified with EDTA for 2 weeks after fixation of the tissues with 10% formalin for 48 h. They were subsequently implanted in paraffin wax and sliced with 4 μm thickness on glass slides. Using hematoxylin and eosin (H&E, Merck), the sliced tissues were stained and observed by an optical microscopy equipped with the Leica Application Suite (Leica Microsystems, Glattbrugg, Switzerland). To grade inflammation and cartilage and bone destruction, histopathological alterations of the rear paws were scored by a 5-point grading system that was previously described (Kang et al., 2020). Synovitis was analyzed on the basis of the criteria which were mentioned previously (Kang et al., 2020).

### Quantitative real time–polymerase chain reaction (qRT-PCR) analysis

Using RNA isolation solution (RNA Bee, Tet-Test Inc., Friendswood, TX, USA), total RNAs were purified from paw connective tissues and followed by synthesis of cDNA with Superscript II reverse transcriptase (Thermo-Fisher Scientific Inc., Cambridge, MA, United States). qRT-PCR was carried out to measure the relative quantities of IL-1β, IL-6 and TNF-α mRNAs using 2× Power SYBR Green (Toyobo Co., Osaka, Japan). The PCR reaction conditions were in this manner: denaturation step (1 min at 95°C), amplifying step (40 cycles of 15 s at 95°C, 15 s at 57°C, and 45 s at 72°C), and melt curve step (15 s at 95°C and 60 s at 60°C). The primer sequences of the cytokines were as follows: IL-1β, sense primer: 5′-GCACA TCAAC AAGAG CTTCA GGCAG-3′ and anti-sense primer: 5′-GCTGC TTGTGAGGTG CTGAT GTAC-3’; IL-6, sense 5′-TTG GGA CTG ATG TTG TTG ACA-3′ and anti-sense 5′-TCA TCG CTG TTG ATA CAA TCA GA-3′; TNF-α, sense 5′-CCT GTA GCC CAC GTC GTA GC-3′ and anti-sense 5′-TTG ACC TCA GCG CTG ACT TG-3′. Additional analyses such as the fluorescence intensity, threshold cycle (Ct), threshold values, and housekeeping genes were performed as previously described (Livak and Schmittgen, 2001).

### Western blotting

As previously described (Kaowinn et al., 2019), proteins from cell lysates were separated using 10% SDS-polyacrylamide gel, and followed by transfer of the proteins onto nitrocellulose membranes. Primary antibodies (1:500–1,000 dilution) and subsequently a horseradish peroxidase conjugated-secondary antibody (1:1,000 dilution) were added to the membrane. Images were visualized with an ImageQuant LAS 4000 Mini (GE Healthcare, Piscataway, NJ, United States) after adding an ECL solution (Thermo Fisher Scientific).

## ELISA

The cytokine concentrations of TNF-α and IL-1β in the culture supernatants from THP-1 cells stimulated with LPS alone, LPS + BMDA, or LPS + DMMA were analyzed with ELISA kits provided by R&D Systems (Minneapolis, MN, United States) in accordance with the manufacturer’s protocols. The level of cytokine-induced neutrophil chemoattractant (CINC)-3 in the agitated distal colon was also quantified with an ELISA kit (R&D Systems) as previously mentioned (Yang et al., 2019).

### Measurement of myeloperoxidase (MPO) activity

The distal colon segments stored at −80°C were minutely chopped up in 1 mL of 0.5% HTAB (pH 6.0) and disrupted using a Polytron homogenizer on ice. The colon tissues were thereafter sonicated for 10 s, freeze-thawed three times, and followed by centrifugation at 14,000 rpm at 4°C. The supernatant (0.1 mL) was then mixed with 2.9 mL of 50 μM PBS enclosing 0.0005% hydrogen peroxide and 0.167 mg/mL odianishidine hydrochloride. The color change of the sample was detected in absorbance at 460 nm using a spectrophotometer (Shimadzu, Kyoto, Japan) for 5 min. The enzyme (MPO) one unit was defined as the decaying 1 μmol of peroxide per minute at 25°C.

### Measurement of AST/ALT

For measurement of serum aspartate aminotransferase (AST) and alanine aminotransferase (ALT) levels, GOT (glutamate-oxaloacetate transaminase for AST) and GPT (glutamate pyruvate transaminase for ALT) reagents (Asan Pharmaceutical, Asan, Korea) were used based on the manufacturer’s protocol. Shortly, after GOT or GPT buffer (100 μL)was added to mouse serum (20 μL), the mixtures were incubated for 30 min or for 60 min at 37°C, respectively. Thereafter, 2,4-dinitrophenylhydrazine colorimetric solution (100 μL, 0.0198%) was added to the mixtures, and followed by the incubation at 25°C for 20 min. The reaction was terminated with 0.4 N NaOH (1 mL) and the absorbance of the reaction color was measured at 505 nm.

### Statistical analysis

All data were described as mean ± standard deviation. Error bars indicate standard deviation. Student’s unpaired *t*-test was utilized for comparing the two groups as a statistical analysis. The significance of the result was set at *p*-value <0.05.

## Results

### BMDA and its derivative DMMA inhibit LPS-induced inflammation *in vitro*

**FIGURE 1:**
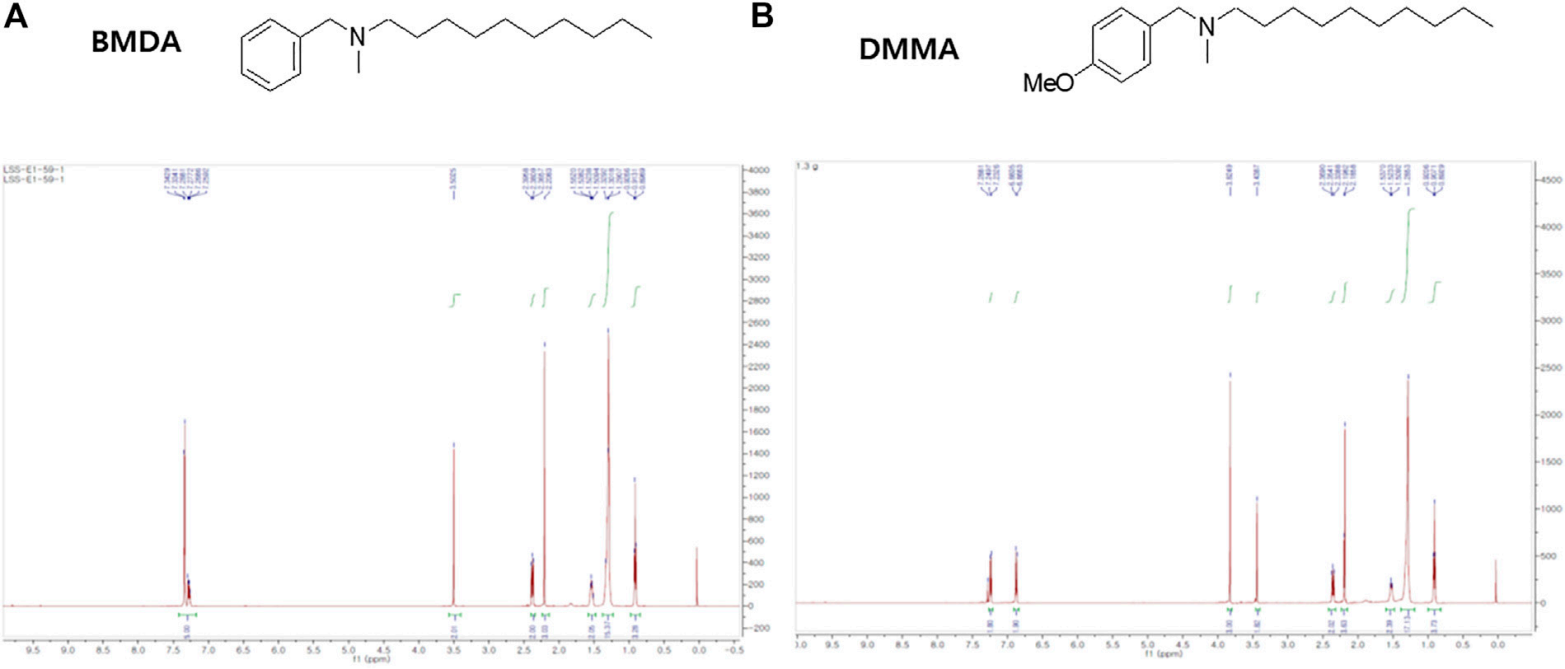
Structure and identification of BMDA and DMMA **(A)** Structure and ^1^H-NMR result after the synthesis of BMDA. **(B)** Structure and ^1^H-NMR result after the synthesis of DMMA.

**FIGURE 2:**
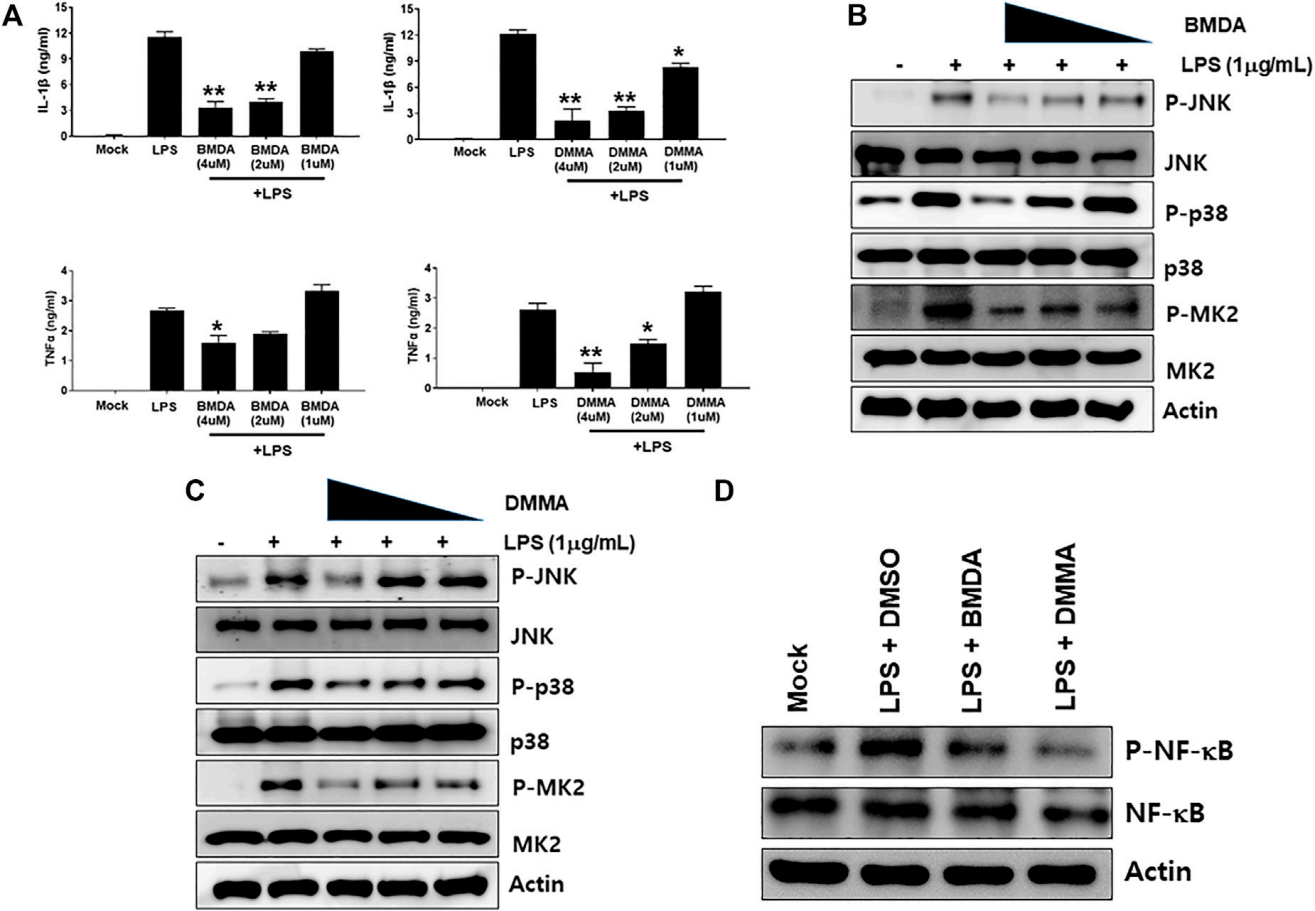
BMDA or DMMA inhibits LPS-induced inflammation through downregulation of JNK/P38 MAPK-MK2 and NF-κB signaling **(A)** After THP-1 cells were pretreated with BMDA (1, 2, and 4 μM), DMMA (1, 2, and 4 μM), or DMSO as a control for 3 h, they were stimulated with LPS (1 μg mL^-1^). The cells were harvested 4 h post-stimulation and subsequently TNF-α and IL-1β cytokines were measured by ELISA. [For IL-1β ELISA, **; *p* < 0.01, LPS vs*.* BMDA (2 or 4 μM)+LPS, LPS vs*.* DMMA(2 or 4 μM)+LPS. *; *p* < 0.05, LPS vs*.* DMMA (1 μM)+LPS. For TNF-α ELISA, **; *p* < 0.01, LPS vs*.* DMMA (4 μM)+LPS. *; *p* < 0.05, LPS vs*.* BMDA (4 μM)+LPS, LPS vs*.* DMMA (2 μM)+LPS]. **(B, C)** THP-1 cells were pretreated with BMDA or DMMA for 3 h and subsequently stimulated with LPS for 1 h. Then, phosphorylation levels of JNK, p38 MAPK, and MK2 in the cells were examined by immunoblotting. **(D)** The cells were pretreated with BMDA (4 μM) or DMMA (4 μM) for 3 h, followed by stimulation of the cells with LPS (1 μg mL^-1^) for 1 h. Then, the cells were harvested and phosphorylation levels of NF-κB p65 protein were detected by immunoblotting.

The chemical structures of BMDA and its derivative DMMA were synthesized by the reductive amination method (Afanasyev et al., 2019) and were confirmed by H^1^-NMR, as presented in Figures 1A, B. Before inquiring that BMDA and DMMA possess anti-inflammation features, we first examined whether these compounds, themselves harbor toxicity to human monocyte THP-1 cells. While LPS (1 μg mL^-1^) induced a slight cell death of THP-1 cells, BMDA and DMMA rather protected the cells from cell death caused by LPS stimulation (Supplementary Figure S1). To examine whether TNF-α and IL-1β cytokine production from THP-1 cells during LPS stimulation are inhibited by pretreatment with BMDA or DMMA, the inflammatory cytokine production was measured in the culture supernatant of the cells by ELISA. BMDA and DMMA clearly diminished TNF-α and IL-1β cytokine production from LPS-stimulated THP-1 cells in a dose-dependent manner (1, 2, and 4 μM) (Figure 2A). To illustrate signaling pathways mediated by inflammatory cytokines such as TNF-α and IL-1β are inactivated by BMDA or DMMA administration, THP-1 cells pretreated with BMDA or DMMA at different concentrations for 3 h were triggered with LPS as a stimulant for 1 h. BMDA and DMMA treatment reduced the phosphorylation levels of JNK and p38MAPK, which are involved in inflammatory cytokines, oxidation, and cellular stress (Figures 2B, C, Supplementary Figures S2A, B). Phosphorylation levels of MK2, a downstream molecule of p38MAPK, also decreased in a similar manner (Figures 2B, C, Supplementary Figure S2C). In addition, as NF-κB p65 protein is known to be involved in inflammatory cytokine production and signaling, phosphorylation levels of NF-κB p65, which is indicating that activated NF-kB protein moves into the nucleus, were examined under the same conditions. During LPS stimulation, pretreatment with BMDA or DMMA inhibited phosphorylation of NF-κB p65 protein (Figure 2D, Supplementary Figure S2D). These results indicate that both BMDA and DMMA possess anti-inflammatory properties *in vitro* through inactivation of JNK/p38MAPK-MK2 and the downregulation of NF-κB.

### Rectal treatment with BMDA or DMMA alleviates DNBS-induced rat colitis

**FIGURE 3:**
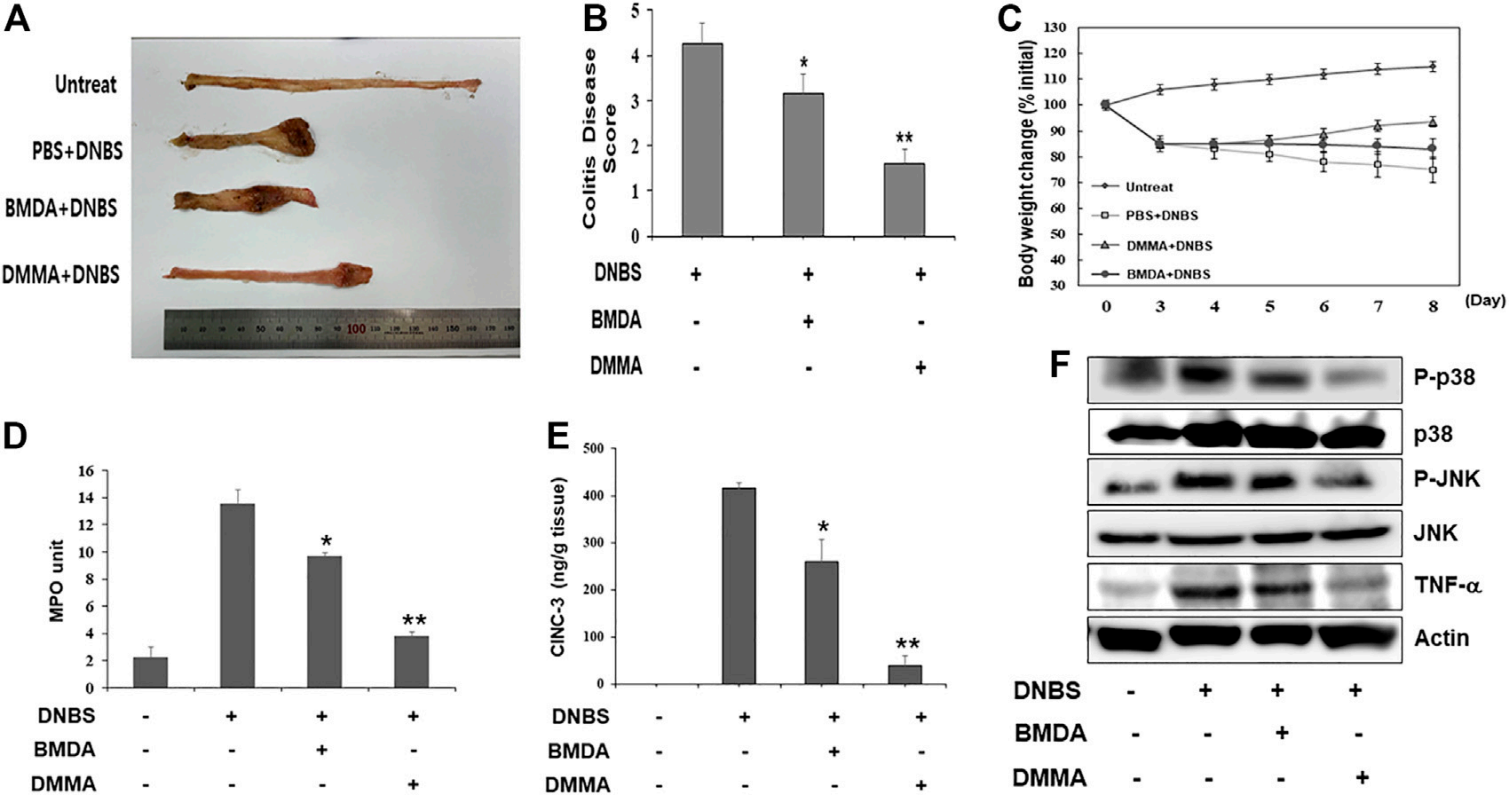
Rectal treatment with BMDA or DMMA relieves DNBS-induced colitis by decreasing inflammatory mediators **(A)** Distal colons of DNBS-induced colitis from rats (*n* = 5 rats per group) treated with BMDA (100 μg, 0.5 mL^−1^ PBS per rat), DMMA (100 μg.0.5 mL^−1^ PBS per rat), or PBS (0.5 mL) as a control for 5 days were photographed. Representative images are shown. **(B)** DNBS-induced colitis in rats treated with BMDA, DMMA, or PBS was evaluated by colon damage score described in Materials and Methods. (**p < 0.01, PBS + DNBS vs*.* DMMA + DNBS. *p < 0.05, PBS + DNBS vs*.* BMDA + DNBS) **(C)** Body weight of rats treated with DNBS for three consecutive days was measured from starting day for rectal administration of BMDA, DMMA or PBS, and the measurement was thereafter kept for 5 days. **(D)** Myeloperoxidase (MPO) activity from inflamed distal colons of rats treated with BMDA, DMMA, or PBS was measured. (**p < 0.01, PBS + DNBS vs*.* DMMA + DNBS. * *p* < 0.05, PBS + DNBS vs*.* BMDA + DNBS) **(E)** Concentration of CINC-3 from inflamed distal colons of rats treated with BMDA, DMMA, or PBS were measured by ELISA. (**; *p* < 0.01, PBS + DNBS vs*.* DMMA + DNBS. * *p* < 0.05, PBS + DNBS vs*.* BMDA + DNBS) **(F)** The distal colons of DNBS-induced colitis from the rats treated with BMDA, DMMA, or PBS were minced and prepared for immunoblotting. Phosphorylation levels of JNK and p38 MAPK, and expression levels of TNF-α in the colons were examined with their specific antibodies.

To explore the anti-inflammatory properties of BMDA and DMMA *in vivo*, a DNBS-induced rat colitis model that mimics IBD was employed. As reported in a previous study (Hong et al., 2012), the DNBS-induced IBD rat model is highly reproducible and an acute inflammatory disease model. In particular, to investigate the direct anti-inflammatory effects of BMDA and DMMA on the cell surface of tissues, we attempted enema treatment of male Sprague Dawley rats with BMDA or DMMA. After rectal administration with DNBS was performed during three consecutive days to induce IBD, BMDA (0.4 mg kg^-1^ body weight) or DMMA (0.4 mg kg^-1^ body weight) diluted with PBS was rectally administered for 5 days. Rats treated with PBS showed very severe colitis inflammation accompanied by hemorrhagic lesions and shorter colon length than untreated rats (Figures 3A, B). BMDA treatment reduced colitis severity but failed to restore colon length (Figures 3A, B). However, the rats treated with DMMA showed reduced colitis inflammation and longer colon length than rats treated with PBS (Figures 3A,B). Interestingly, DMMA treatment resulted in improved colitis inflammation and longer colon length than BMDA treatment (Figures 3A, B). However, BMDA or DMMA administration did not fully recover body weight as seen in untreatment group (Figure 3C) Consistent with the colitis score results, DMMA treatment resulted in lower activity and concentration of inflammatory mediators, such as MPO activity and CINC-3 in the colonic tissues than those from BMDA or PBS treatment (Figures 3D, E). In addition, when signaling molecules involved in inflammation were examined in the colonic tissues, phosphorylation levels of JNK and p38MAPK and expression levels of TNF-α were decreased during BMDA or DMMA administration (Figure 3F). Based on these results, both BMDA and DMMA treatment exhibited anti-inflammatory effects on DNBS-induced acute colitis, and furthermore, DMMA showed greater therapeutic efficacy than BMDA for the treatment of DNBS-induced rat colitis.

### Oral administration with BMDA or DMMA alleviates collagen-induced RA

**FIGURE 4:**
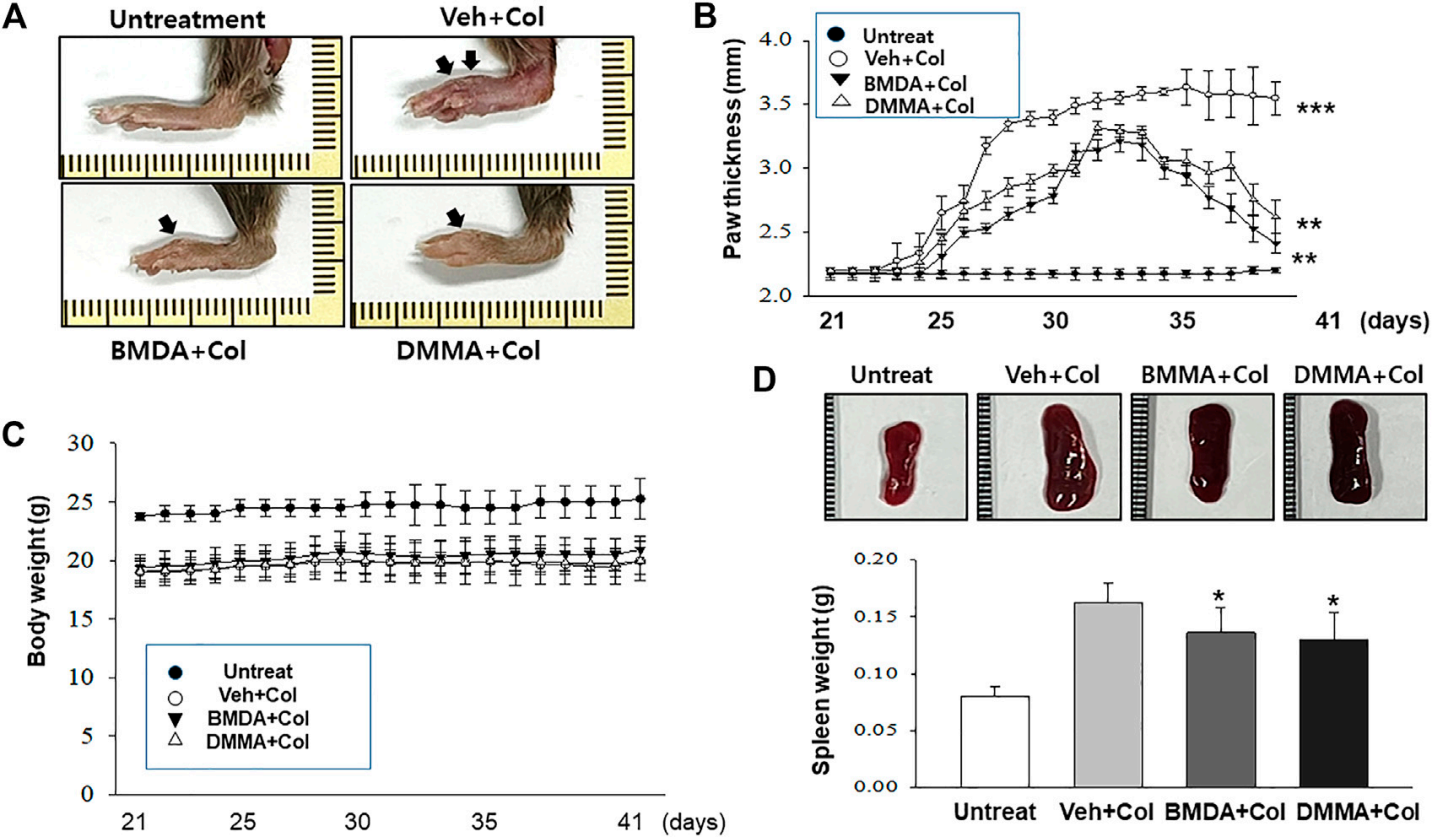
Oral administration of BMDA or DMMA reduces collagen-induced RA **(A)** RA was induced in DBA mice by collagen. Hind paw tissues from the mice (*n* = 6 mice per group) orally treated with BMDA (0.5 mg0.2 mL^−1^ corn oil per mouse), DMMA (0.5 mg0.2 mL^-1^corn oil per mouse), or corn oil (0.2 mL) as a control were photographed. Representative images are shown. **(B)** Thickness of hind paw from the animals were measured from the first day to 21 days after sensitization of collagen and IFA every other day using a caliper. Vehicle (Veh) indicates corn-oil. (***; *p* < 0.001, untreatment vs*.* Veh + Collagen (Col), **; *p* < 0.01, Veh + Collagen(Col) vs*.* BMDA + Col, Veh + Col vs*.* DMMA + Col) **(C)** Body weight of the animal was also measured when hind paw thickness was measured. **(D)** Spleen size and weight of the animals were measured 21 days post-sensitization of collagen and IFA. (**; *p* < 0.01, untreatment vs*.* Veh + Collagen (Col), *; *p* < 0.05, Veh + Collagen(Col) vs*.* BMDA + Col, Veh + Col vs*.* DMMA + Col).

**FIGURE 5:**
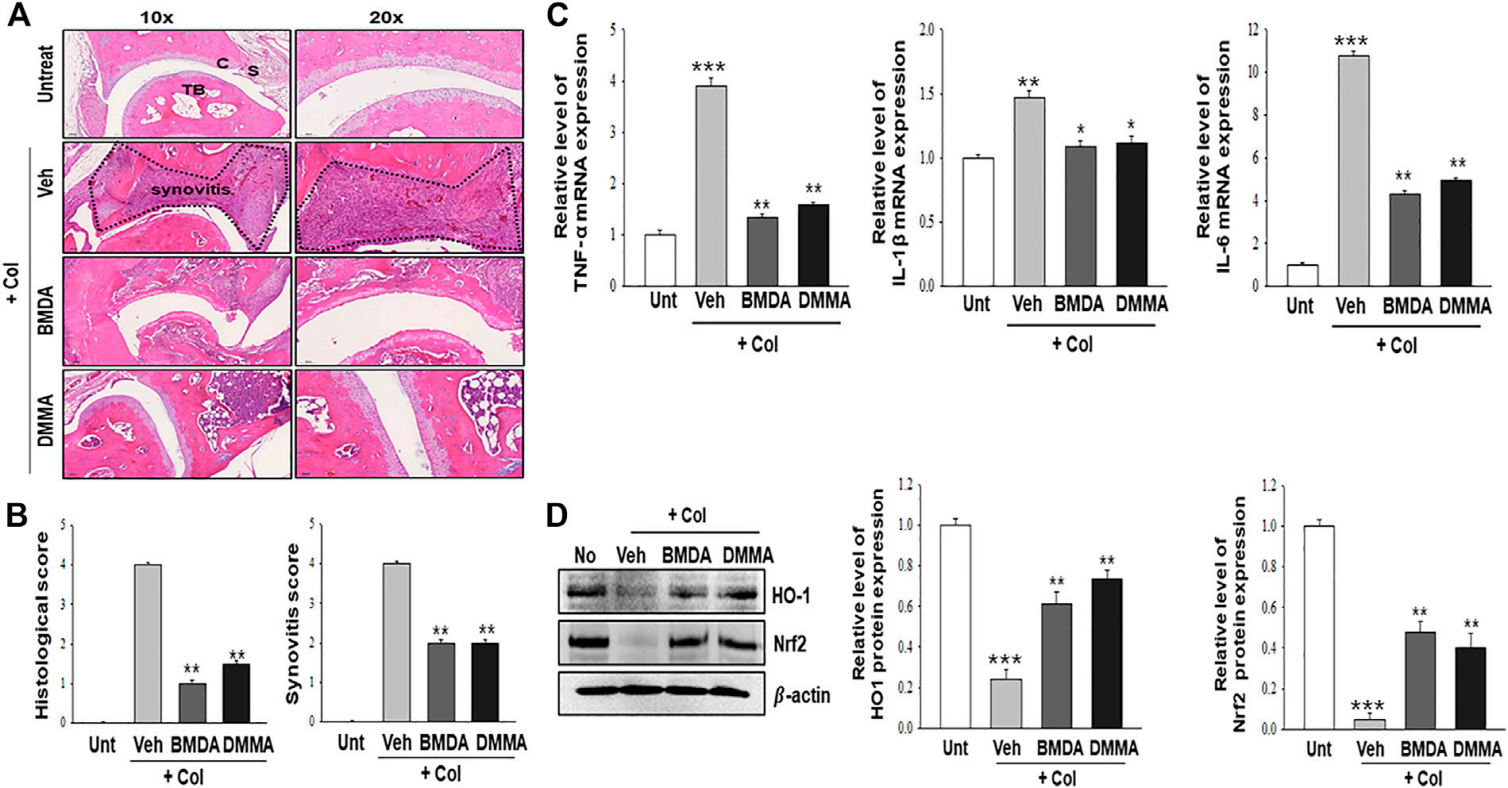
BMDA or DMMA-mediated reduction of severity is attributed to decrease of inflammatory cytokine transcripts and enhancement of anti-oxidation protein expression **(A, B)** Histopathological analysis on hind paw tissues of the animals orally treated with BMDA, DMMA, or corn oil as a control was performed with hematoxylin and eosin staining 21 days post-sensitization of collagen and IFA. Synovitis score was evaluated as described previously (Kang et al., 2020). **(C)** cartilage, S: synovium, TB: trabecular bone. (**; *p* < 0.01, Veh + Col vs*.* BMDA + Col, Veh + Col vs*.* DMMA + Col) **(C)** Transcript levels of inflammatory cytokines such as TNF-α, IL-1β, and IL-6 were measured by qRT-PCR from the paw tissues of the animals. (For TNF-α and IL-6 qRT-PCR, ***; *p* < 0.001, untreatment vs*.* Veh + Col, **; *p* < 0.01, Veh + Col vs*.* BMDA + Col, Veh + Col vs*.* DMMA + Col. For IL-1β qRT-PCR, **; *p* < 0.01, untreatment vs*.* Veh + Col, *; *p* < 0.05, Veh + Col vs*.* BMDA + Col, Veh + Col vs*.* DMMA + Col) **(D)** Expression of anti-oxidation proteins such as HO1 and Nrf2 was examined by immunoblotting and the relative expression of the proteins was analyzed after scanning using AlphaView, version 3.2.2 (Cell Biosciences Inc., Santa Clara, CA, United States). (For HO1 protein, ***; *p* < 0.001, untreatment vs*.* Veh + Col, **; *p* < 0.01, Veh + Col vs*.* BMDA + Col, Veh + Col vs*.* DMMA + Col. For Nrf2 protein, ***; *p* < 0.001, untreatment vs*.* Veh + Col, **; *p* < 0.01, Veh + Col vs*.* BMDA + Col, Veh + Col vs*.* DMMA + Col).

**FIGURE 6:**
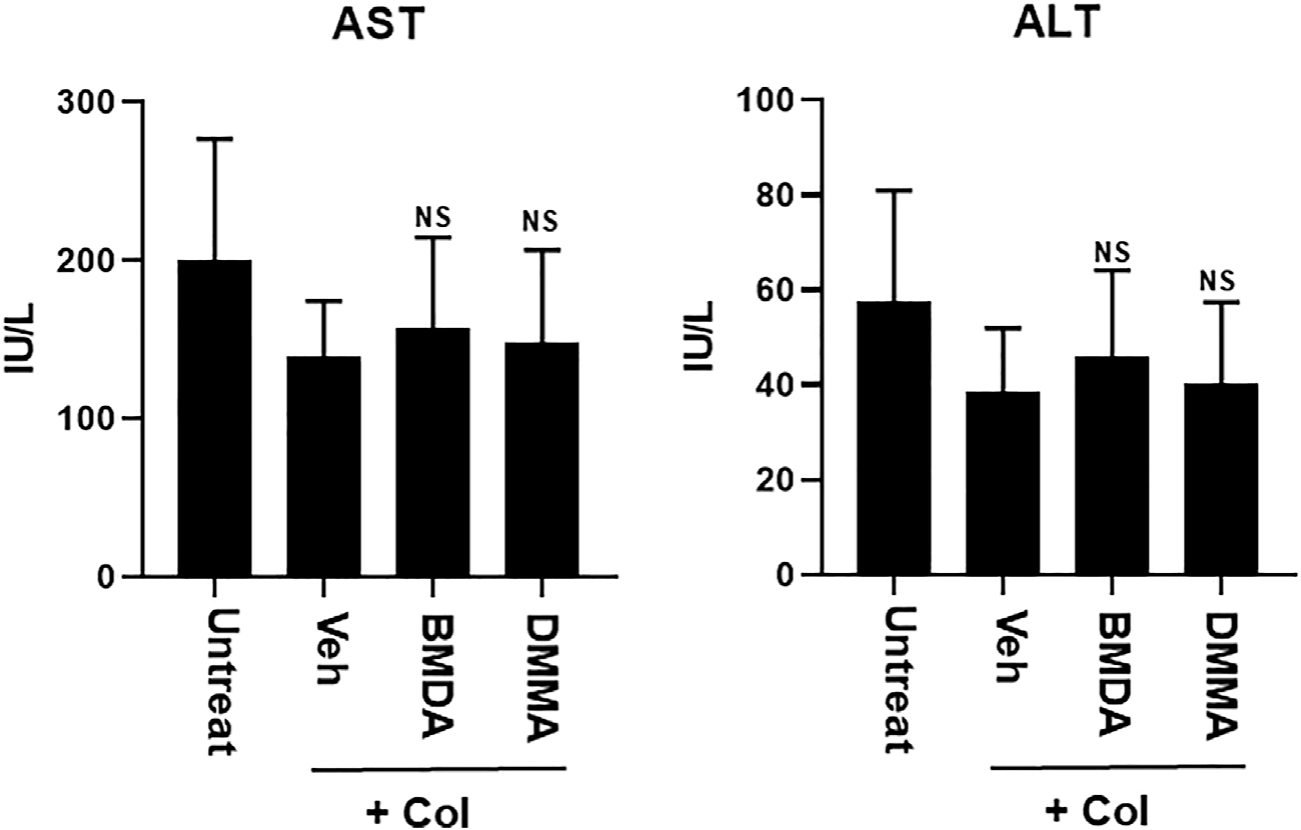
Oral treatment with BMDA or DMMA does not affect liver toxicity in the RA-carrying mice. AST and ALT levels were measured in the sera of RA-carrying mice that were orally treated with BMDA, DMMA, or corn oil 21 days post-sensitization of collagen and IFA. NS indicates not significant (ns; Veh + Col vs. BMDA + Col, Veh + Col vs. DMMA + Col).

As we demonstrated that both BMDA and DMMA have anti-inflammatory effects in an acute inflammatory disease model, such as DNBS-induced rat colitis (Figures 3A–F), we wondered whether these small molecules could alleviate chronic inflammatory diseases, such as collagen-induced RA. We were also curious whether oral administration of BMDA or DMMA could exert anti-inflammatory effects in collagen-induced RA. DBA/1 mice were consecutively treated with a mixture of CFA and type 2 bovine collagen (1:1), followed by IFA and the collagen (1:1) 18 days later. Three days after the second co-administration, the mice were separated into three groups: vehicle, BMDA, and DMMA. BMDA or DMMA diluted with corn oil was orally administered to the animals every day for 21 days after grouping. The vehicle group treated with corn oil showed a gradual increase in paw thickness (from ∼2.1 to ∼3.4 mm) until 30 days after the first immunization and maintained paw thickness (from ∼3.4 to ∼3.6 mm) thereafter (Figures 4A, B). BMDA or DMMA treatment reduced the progression of inflammation in the paw compared to vehicle treatment, but inflammation in the paw reached its maximum around 35 days after the first immunization and then drastically decreased until 42 days (BMDA; ∼2.5 mm, DMMA; ∼2.8 mm) (Figures 4A, B). Interestingly, BMDA exerted slightly greater anti-inflammatory efficacy than DMMA, which was the opposite in the DNBS-induced rat colitis model (Figures 4A, B). BMDA and DMMA treatment failed to completely recover the collagen-induced loss of body weight (Figure 4C). The protocol for collagen-induced RA increased the spleen size and weight in the DBA/1 mice, but BMDA or DMMA treatment inhibited the increase in the spleen size and weight (Figure 4D). Furthermore, staining of the hind paw tissues in the vehicle-treated mice with hematoxylin-eosin showed severe synovitis, but synovitis was clearly reduced in the hind paw tissues of BMDA- or DMMA-treated mice (Figures 5A, B). When mRNA levels of inflammatory cytokines were examined in the hind paw tissues of the animals, BMDA or DMMA treatment diminished transcript levels of IL-1β, IL-6, and TNF-α in the tissues compared with those in the tissues from vehicle-treated animals (Figure 5C). Interestingly, when the expression levels of anti-oxidation proteins such as HO1 and Nrf2 were examined, BMDA or DMMA treatment inhibited collagen-induced decrease of HO1 and Nrf2 protein levels in the tissues of vehicle-treated animals (Figure 5D). Moreover, as we were curious about the toxicity of BMDA and DMMA, the levels of AST and ALT, an indicator of liver toxicity, were measured in the sera of animals treated with vehicle, BMDA, or DMMA. We found that the levels of AST and ALT in the mice treated with BMDA or DMMA were similar to those in the animals treated with vehicle (Figure 6), indicating that neither BMDA nor DMMA induced toxicity in the liver of animals orally treated with BMDA or DMMA for 3 weeks. Based on these results, oral administration of BMDA and DMMA exhibited anti-inflammatory and anti-oxidative effects in collagen-induced RA, a chronic inflammatory disease model.

## Discussion

This study was initiated to explore whether BMDA and DMMA possess therapeutic effects on both acute and chronic inflammatory diseases. In addition, we wondered whether these small molecules exert the anti-inflammatory activity irrespective of a route administration. To answer these questions, we thus employed DNBS-induced colitis representing an acute inflammatory disease model and collagen-induced RA displaying a relatively chronic inflammatory disease model. Moreover, we implemented the direct delivery of BMDA and DMMA to epithelial cells of colorectal colon in the rats bearing colitis and oral administration of them to mice suffering RA in order to remedy the inflammatory diseases irrespective of a route administration.

We report for the first time that BMDA and its derivative, DMMA, exert anti-inflammatory effects on DNBS-induced colitis and collagen-induced RA disease, which could be attributed to the downregulation of p38MAPK-MK2 inflammation and suppression of the NF-κB signaling pathway. As a consequence, BMDA or DMMA administration reduced expression levels of inflammatory cytokines such as IL-1β and TNF-α in LPS-stimulated THP-1 cells *in vitro* and in tissues from DNBS-induced colitis and collagen-induced arthritis *in vivo*. However, a careful examination revealed BMDA showing a slightly better therapeutic effect than DMMA on collagen-induced RA, but the opposite result was observed in DNBS-induced colitis. To explain these findings, we propose that the structure of DMMA itself is more effective in suppressing inflammatory progression in DNBS-induced colitis. However, orally administered DMMA might be converted to another form or be metabolized during delivery from the mouth to the small intestine and then to the blood circulation, which could be a cause for the low effectiveness on the pathological progression of collagen-induced RA. In a future study, we would explore whether the structure of BMDA or DMMA should be examined in the sera after oral treatment.

Allicin, a key constituent of garlic, decreases LPS-induced oxidative stress and NF-κB activity through the Nrf2 anti-oxidative signaling pathway, leading to the prevention of endothelial cell apoptosis (Pandey et al., 2020). A recent study also reported that allicin treatment increased regulatory T cell populations but decreased the number of Th17 cells in collagen-induced arthritis through the upregulation of MEKK2 expression (Zhang and Gong, 2021). Diallyl trisulfide also reduced arthritis induced by collagen through inhibition of the Wnt and NF-κB signaling pathways (Liang et al., 2019). Similar to allicin and diallyl trisulfide, both BMDA and DMMA diminished NF-κB expression and translocation into the nucleus. Because spleen weight and size were reduced after oral treatment with BMDA or DMMA in collagen-induced DBA/1 mice compared to those in vehicle-treated mice, we might set up the hypothesis that BMDA or DMMA administration suppresses the proliferation of T and B cell populations reacting with RA-relevant autoantigens. A study to determine whether BMDA or DMMA treatment inhibits proliferation and activation of the T17 population, which is known to play a critical role in RA pathogenesis, remains to be undertaken as future work.

Interestingly, allicin and diallyl trisulfide have also shown anticancer activity (Pandey et al., 2020) (Kim et al., 2016). Further investigation has demonstrated that these compounds reduce antioxidant protein levels, such as Nrf2 and HO1, leading to the upregulation of ROS, which eventually results in the induction of apoptosis or autophagy (Zhang and Yang, 2019). These results indicate that the same compounds could induce the upregulation or downregulation of antioxidant protein levels, depending on the experimental setup. Thus, we carefully interpret these results from the viewpoint of garlic compound concentration, treatment time, and cell line type. Additionally, our previous study reported that treatment with a high concentration of BMDA (40 mg kg^-1^ body weight) inhibited the development of tumors *in vivo* (Kaowinn et al., 2018) compared to the concentration of BMDA (0.4 mg kg^-1^ body weight) in DNBS-induced rat colitis and its concentration (20 mg kg^-1^ body weight) in a collagen-induced RA model. Therefore, further studies are needed to optimize the concentrations of BMDA and DMMA for clinical application in IBD and RA.

## Conclusion

Both BMDA and DMMA possess anti-inflammatory properties by downregulating the JNK/p38MAPK-MK2 and NF-κB inflammatory signaling pathways *in vitro*. In addition, treatment with these compounds reduce severity of inflammation in DNBS-induced rat colitis and collagen-induced RA mouse model. These discoveries propose that BMDA and DMMA could be developed as novel remedial drugs for curing IBD and RA.

## Data Availability

The original contributions presented in the study are included in the article/Supplementary Material, further inquiries can be directed to the corresponding authors.

## Abbreviations

AST, aspartate aminotransferase; ALT, alanine aminotransferase; BMDA, N-benzyl-N-methyldecan-1-amine; DMMA, [decyl-(4-methoxy-benzyl)-methyl-amine; DNBS, 2,4-dinitrobenzenesulfonic acid; HO1, heme oxygenase1; IBD, inflammatory bowel disease; LPS, Lipopolysaccharide; Nrf2, nuclear factor erythroid-related factor; RA, rheumatoid arthritis; ROS, reactive oxygen species.

## References

[B1] Abraham C. Cho J. H. (2009). Inflammatory bowel disease. N. Engl. J. Med. 361, 2066–2078. 10.1056/NEJMra0804647 19923578 PMC3491806

[B2] Afanasyev O. I. Kuchuk E. Usanov D. L. Chusov D. (2019). Reductive amination in the synthesis of pharmaceuticals. Chem. Rev. 119, 11857–11911. 10.1021/acs.chemrev.9b00383 31633341

[B3] Demoruelle M. K. Deane K. D. (2012). Treatment strategies in early rheumatoid arthritis and prevention of rheumatoid arthritis. Curr. Rheumatol. Rep. 14, 472–480. 10.1007/s11926-012-0275-1 22773387 PMC3616381

[B4] Hong S. Yum S. Yoo H. J. Kang S. Yoon J. H. Min D. (2012). Colon-targeted cell-permeable NFκB inhibitory peptide is orally active against experimental colitis. Mol. Pharm. 9, 1310–1319. 10.1021/mp200591q 22428658

[B5] Huang J. Fu X. Chen X. Li Z. Huang Y. Liang C. (2021). Promising therapeutic targets for treatment of rheumatoid arthritis. Front. Immunol. 12, 686155. 10.3389/fimmu.2021.686155 34305919 PMC8299711

[B6] Hyrich K. L. Silman A. J. Watson K. D. Symmons D. P. (2004). Anti-tumour necrosis factor alpha therapy in rheumatoid arthritis: An update on safety. Ann. Rheum. Dis. 63, 1538–1543. 10.1136/ard.2004.024737 15242866 PMC1754871

[B7] Jeong J. W. Park S. Park C. Chang Y. C. Moon D. O. Kim S. O. (2014). N-benzyl-N-methyldecan-1-amine, a phenylamine derivative isolated from garlic cloves, induces G2/M phase arrest and apoptosis in U937 human leukemia cells. Oncol. Rep. 32, 373–381. 10.3892/or.2014.3215 24859825

[B8] Kang E. J. Kim H. J. Choi J. H. Noh J. R. Kim J. H. Lee I. B. (2020). Humulus japonicus extract ameliorates collagen-induced arthritis in mice through regulation of overall articular inflammation. Int. J. Mol. Med. 45, 417–428. 10.3892/ijmm.2019.4417 31894253 PMC6984789

[B9] Kang L. J. Kwon E. S. Lee K. M. Cho C. Lee J. I. Ryu Y. B. (2018). 3'-Sialyllactose as an inhibitor of p65 phosphorylation ameliorates the progression of experimental rheumatoid arthritis. Br. J. Pharmacol. 175, 4295–4309. 10.1111/bph.14486 30152858 PMC6240131

[B10] Kaowinn S. Kaewpiboon C. Kim J. E. Lee M. R. Hwang D. Y. Choi Y. W. (2018). N-Benzyl-N-methyl-dodecan-1-amine, a novel compound from garlic, exerts anti-cancer effects on human A549 lung cancer cells overexpressing cancer upregulated gene (CUG)2. Eur. J. Pharmacol. 841, 19–27. 10.1016/j.ejphar.2018.09.035 30287155

[B11] Kaowinn S. Oh S. Moon J. Yoo A. Y. Kang H. Y. Lee M. R. (2019). CGK062, a small chemical molecule, inhibits cancer upregulated gene 2-induced oncogenesis through NEK2 and β-catenin. Int. J. Oncol. 54, 1295–1305. 10.3892/ijo.2019.4724 30968157 PMC6411349

[B12] Kim S. H. Kaschula C. H. Priedigkeit N. Lee A. V. Singh S. V. (2016). Forkhead box Q1 is a novel target of breast cancer stem cell inhibition by diallyl trisulfide. J. Biol. Chem. 291, 13495–13508. 10.1074/jbc.M116.715219 27129776 PMC4919436

[B13] Kim W. Kim S. Ju S. Lee H. Jeong S. Yoo J. W. (2019). Colon-Targeted delivery facilitates the therapeutic switching of sofalcone, a gastroprotective agent, to an anticolitic drug via Nrf2 activation. Mol. Pharm. 16, 4007–4016. 10.1021/acs.molpharmaceut.9b00664 31386809

[B14] Lee H. H. Jeong J. W. Hong S. H. Park C. Kim B. W. Choi Y. H. (2018). Diallyl trisulfide suppresses the production of lipopolysaccharide-induced inflammatory mediators in BV2 microglia by decreasing the NF-κB pathway activity associated with toll-like receptor 4 and CXCL12/CXCR4 pathway blockade. J. Cancer Prev. 23, 134–140. 10.15430/JCP.2018.23.3.134 30370258 PMC6197846

[B15] Liang J. J. Li H. R. Chen Y. Zhang C. Chen D. G. Liang Z. C. (2019). Diallyl Trisulfide can induce fibroblast-like synovial apoptosis and has a therapeutic effect on collagen-induced arthritis in mice via blocking NF-κB and Wnt pathways. Int. Immunopharmacol. 71, 132–138. 10.1016/j.intimp.2019.03.024 30897500

[B16] Livak K. J. Schmittgen T. D. (2001). Analysis of relative gene expression data using real-time quantitative PCR and the 2(-Delta Delta C(T)) Method. Methods 25, 402–408. 10.1006/meth.2001.1262 11846609

[B17] Noss E. H. Brenner M. B. (2008). The role and therapeutic implications of fibroblast-like synoviocytes in inflammation and cartilage erosion in rheumatoid arthritis. Immunol. Rev. 223, 252–270. 10.1111/j.1600-065X.2008.00648.x 18613841

[B18] Pandey N. Tyagi G. Kaur P. Pradhan S. Rajam M. V. Srivastava T. (2020). Allicin overcomes hypoxia mediated cisplatin resistance in lung cancer cells through ROS mediated cell death pathway and by suppressing hypoxia inducible factors. Cell Physiol. Biochem. 54, 748–766. 10.33594/000000253 32809300

[B19] Pithadia A. B. Jain S. (2011). Treatment of inflammatory bowel disease (IBD). Pharmacol. Rep. 63, 629–642. 10.1016/s1734-1140(11)70575-8 21857074

[B20] Rath T. Billmeier U. Ferrazzi F. Vieth M. Ekici A. Neurath M. F. (2018). Effects of anti-integrin treatment with vedolizumab on immune pathways and cytokines in inflammatory bowel diseases. Front. Immunol. 9, 1700. 10.3389/fimmu.2018.01700 30131801 PMC6090141

[B21] Smolen J. S. Landewé R. Bijlsma J. Burmester G. Chatzidionysiou K. Dougados M. (2017). EULAR recommendations for the management of rheumatoid arthritis with synthetic and biological disease-modifying antirheumatic drugs: 2016 update. Ann. Rheum. Dis. 76, 960–977. 10.1136/annrheumdis-2016-210715 28264816

[B23] Triantafillidis J. K. Merikas E. Georgopoulos F. (2011). Current and emerging drugs for the treatment of inflammatory bowel disease. Drug Des. Devel Ther. 5, 185–210. 10.2147/DDDT.S11290 PMC3084301 21552489

[B24] Uhlig H. H. Powrie F. (2018). Translating Immunology into therapeutic concepts for inflammatory bowel disease. Annu. Rev. Immunol. 36, 755–781. 10.1146/annurev-immunol-042617-053055 29677472

[B25] Yang Y. Kim W. Kim D. Jeong S. Yoo J. W. Jung Y. (2019). A colon-specific prodrug of metoclopramide ameliorates colitis in an experimental rat model. Drug Des. Devel Ther. 13, 231–242. 10.2147/dddt.s185257 PMC6312693 30643389

[B26] Zhang M. Pan H. Xu Y. Wang X. Qiu Z. Jiang L. (2017). Allicin decreases lipopolysaccharide-induced oxidative stress and inflammation in human umbilical vein endothelial cells through suppression of mitochondrial dysfunction and activation of Nrf2. Cell Physiol. Biochem. 41, 2255–2267. 10.1159/000475640 28456799

[B27] Zhang Q. Yang D. (2019). Allicin suppresses the migration and invasion in cervical cancer cells mainly by inhibiting NRF2. Exp. Ther. Med. 17, 1523–1528. 10.3892/etm.2018.7104 30783417 PMC6364242

[B28] Zhang Y. Gong Y. (2021). Allicin regulates Treg/Th17 balance in mice with collagen-induced arthritis by increasing the expression of MEKK2 protein. Food Sci. Nutr. 9, 2364–2371. 10.1002/fsn3.2034 34026055 PMC8116865

[B29] Zhang Y. Liu X. Ruan J. Zhuang X. Zhang X. Li Z. (2020). Phytochemicals of garlic: Promising candidates for cancer therapy. Biomed. Pharmacother. 123, 109730. 10.1016/j.biopha.2019.109730 31877551

