# Supplementary material for: N-benzyl-N-methyldecan-1-amine and its derivative mitigate 2,4- dinitrobenzenesulfonic acid-induced colitis and collagen-induced rheumatoid arthritis
Source: Front Pharmacol. 2023 Apr 20;14:1095955. doi: 10.3389/fphar.2023.1095955 (PMC10157284; doi:10.3389/fphar.2023.1095955)
Supplement: Supplementary file 1 [file DataSheet1.docx]

**N-benzyl-N-methyldecan-1-amine and its derivative mitigate 2,4- dinitrobenzenesulfonic acid-induced colitis and collagen-induced rheumatoid arthritis**

**Supplementary figure 1**


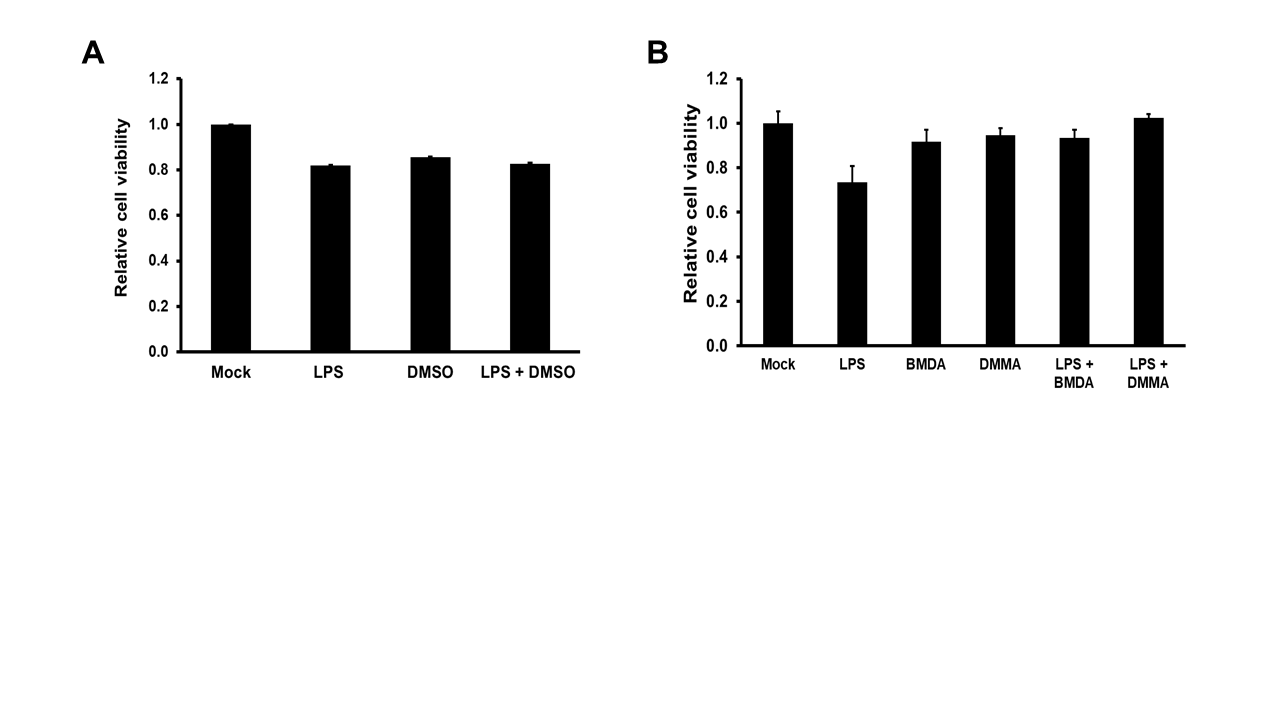


**THP-1 cell viability during LPS, LPS+BMDA, or LPS+DMMA treatment**

(A) After THP-1 cells were treated with LPS (1μg.mL^-1^) alone, DMSO (10 μL.mL^-1^) alone , and LPS (1μg.mL^-1^)+DMSO (10 μL.mL^-1^) for 4 h, the cell viability was measured with MTT assay. (B) After THP-1 cells were pre-treated with BMDA (4 μM) and DMMA (4 μM) for 3 h, the cells were stimulated with LPS (1μg.mL^-1^) for 21 h. Then, the cell viability was measured with MTT assay.

**Supplementary figure 2**


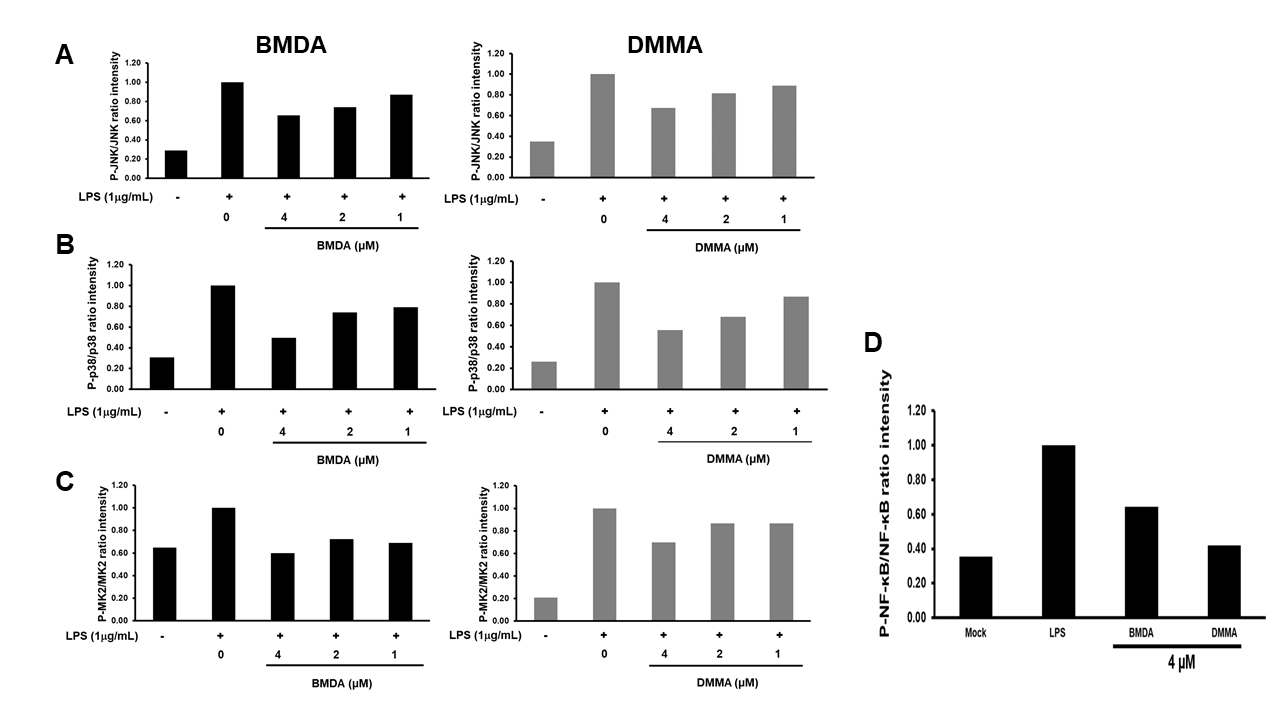


**Phosphorylation levels of JNK, p38MAPK, MK2 and NF-κB in the THP-1 cells during LPS, LPS+BMDA or LPS+DMMA treatment**

(A-D) THP-1 cells were pre-treated with BMDA (4 μM) or DMMA (4 μM) for 3 h and then stimulated with LPS for 1 h. The cells were harvested, and the cell lysates were prepared, followed by immunoblotting. Phosphorylated JNK(A), p38MAPK(B), MK2(C) and NF-κB(D) were detected with their specific antibodies. After scanning of the band intensity, the phosphorylation levels were analyzed with Image J program (NIH).
